# Guyana’s budget 2022: Potential for a boost to planetary health in the Caribbean region

**DOI:** 10.7189/jogh.12.03022

**Published:** 2022-06-04

**Authors:** Sandeep B Maharaj, Samuel Ramsewak, Darleen Y Franco, Amrica Ramdass

**Affiliations:** 1 Associaciate Dean Distance Education, Planning and Projects, Faculty of Medical Sciences, The University of the West Indies, St. Augustine Campus, St. Augustine, Trinidad and Tobago; 2 Global Outreach Fellow Planetary Health Alliance, Boston, Massachusetts, USA; 3 Department of Clinical Surgical Sciences, The University of the West Indies St. Augustine Campus, St. Augustine, Trinidad and Tobago; 4 North West Regional Health Authority, Port of Spain Trinidad and Tobago; 5 Dean’s Office Faculty of Medical Sciences St. Augustine Campus, St Augustine, Trinidad and Tobago

The Caribbean region is continuously threatened by a storm of events that can pose severe threats to human health. The archipelago and its English-speaking continental partners (Belize, Guyana, and Suriname) are recognized as one of the world’s biodiversity hotspots, which are threatened by the impacts of climate change such as rising sea levels, intensifying hurricanes and droughts, mass coral bleaching events, and a potential shortage of water and food storage. While tackling these challenges, the region is also confounded by the short and long-term dangers of the COVID-19 pandemic and its global public health challenge [1]. In this context, the need to better understand the relationship between human health and the environment is urgent, and so, a call to action is drastically needed to address planetary health issues. From a fiscal policy standpoint, Guyana is leading the way, as evidenced in its 2022 budget statement which lays a roadmap for the type of transformation required to enhance a sustainable system.

The Republic of Guyana (population 787 000) is situated in the northeast of the South American continent, with a coastline on the North Atlantic Ocean and with international borders to Brazil, Suriname, and Venezuela. It contributes maritime borders with Trinidad and Tobago and Barbados. Most of the country is covered in tropical rainforests and it holds rich reserves of bauxite, gold, and timber [2]. Guyana is considered as one of the ‘greenest’ countries in the world which has an estimated forest area of 18.4 million square miles of forest area, covering about 87% of the country’s total land area. Within the Caribbean and the South American region, Guyana has a good reputation of forest governance, established mainly by political will and efforts to finance conservation and preservation of its unexplored rainforest. Deforestation rates are among the lowest in the world and Guyana is one of the only four countries worldwide to have sustained a High Forest Low Deforestation (HFLD) state [3]. The country's 2022 budget, themed “Steadfast against All Challenges, Resolute in Building Our One Guyana”, with a value of US$2.65 billion, is 44.3% larger than in 2021. Real Gross Domestic Product (GDP) is expected to grow by 47.5%, a rate that no other country is anticipated to achieve in 2022. Despite the fact that Guyana’s monetary windfall is linked to newly discovered and massive offshore hydrocarbon deposits, the Agenda outlined plans in the budget for a Low Carbon Development Strategy (LCDS), which shapes a new low carbon economy through incentives that value the world's ecosystem services and promote them as a vital feature of a new model of global development with sustainability at its core.

**Figure:**
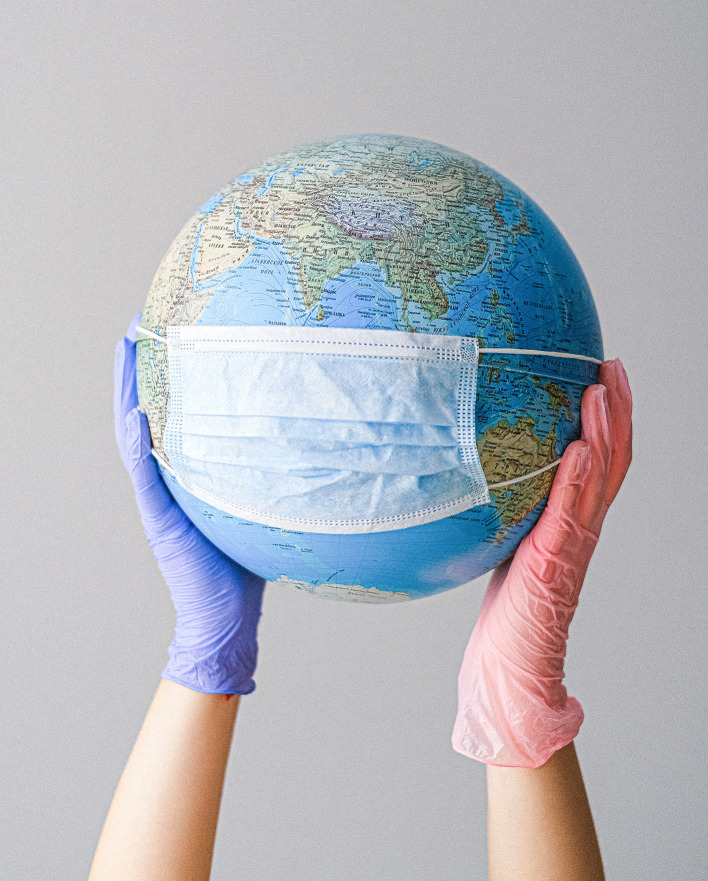
Photo: Making planetary health a reality, require balancing environmental impact against economic gain. Source: https://www.pexels.com/photo/hands-with-latex-gloves-holding-a-globe-with-a-face-mask-4167544/). Permission for use not needed (open-source photo).

From its inception in 2009, the LCDS has been fundamental in shaping the future of Guyana’s green economy efforts. In order to accomplish the objectives outlined in Guyana’s LCDS, international action was sought from partners who shared the country’s vision. Guyana and Norway agreed to work together to establish a model on how progress on economic incentives for its forested areas could be made. An agreement was signed by the two countries where Norway would provide Guyana with result-based payments for climate change, the protection of biodiversity, and the enhancement of sustainable development. From 2009-2015, Guyana earned US$212.52 million in payments to be invested in the LCDS, which funded projects such as sustainable forest use and development, clean energy, and indigenous peoples’ rights and development. The 2009 LCDS was updated in 2013, overseen by a Multi-Stakeholder Steering Committee (MSSC) which combined national plans for investment with calls for international action on climate change [4].

The strategy, which sets out Guyana’s plans for low carbon growth, with reduced exploitation of its forest reserves and with affiliated benefits for climate change mitigation, is a boost towards Planetary Health in the Caribbean. Guyana's new LCDS 2030 will recognize seven solutions that balance the need for healthy communities, from creating new incentives for a low-carbon economy to protecting against climate change and biodiversity loss. Guyana is committed to advancing the LCDS and collaborating with international partners to expand their work and payment for forest climate and ecosystem services. The Government also intends to use revenues from oil production to boost other sectors of the economy, such as agriculture and infrastructure [5].

Since a changing climate is associated with altering the pattern of disease, threats to plant, animal and human life, water insecurity, and increasing temperatures, the Government’s addressing of these and other issues will ultimately repair these dilemmas and aid in the protection of a healthy planet. It is also important to note that though putting people at the forefront of development is crucial, a lot more needs to be accomplished, such as the move to solar and hydroelectric energy. These forms of energy are recognised as contributors to a more sustainable environment for all [6].

A budget which focuses on environmental sustainability is expected to bring benefits to the people of Guyana, its neighbours, and the world at large.
